# Biopackaging Potential Alternatives: Bioplastic Composites of Polyhydroxyalkanoates and Vegetal Fibers

**DOI:** 10.3390/polym14061114

**Published:** 2022-03-10

**Authors:** Natalia Gómez-Gast, Ma Del Rocío López Cuellar, Berenice Vergara-Porras, Horacio Vieyra

**Affiliations:** 1 Tecnologico de Monterrey, Escuela de Ingeniería y Ciencias, Carretera Lago de Guadalupe 3.5, Colonia Margarita Maza de Juárez, Atizapán de Zaragoza 52926, Mexico; a00354363@tec.mx (N.G.-G.); vergarabp@gmail.com (B.V.-P.); 2 Cuerpo Académico de Biotecnología Agroalimentaria (CABA), Institute of Food and Agricultural Sciences (ICAp), Autonomous University of Hidalgo State (UAEH), Av. Universidad Km. 1, Ex-Hda. De Aquetzalpa AP 32, Tulancingo de Bravo 43600, Mexico; marocio_lopez@uaeh.edu.mx; 3 Tecnologico de Monterrey, Escuela de Ingeniería y Ciencias, Eduardo Monroy Cardenas 2000, San Antonio Buenavista, Toluca de Lerdo 50110, Mexico

**Keywords:** polyhydroxyalkanoates, fibers, mechanical properties, biodegradability, packaging, patents

## Abstract

Initiatives to reduce plastic waste are currently under development worldwide. As a part of it, the European Union and private and public organizations in several countries are designing and implementing regulations for single-use plastics. For example, by 2030, plastic packaging and food containers must be reusable or recyclable. In another approach, researchers are developing biopolymers using biodegradable thermoplastics, such as polyhydroxyalkanoates (PHAs), to replace fossil derivatives. However, their production capacity, high production costs, and poor mechanical properties hinder the usability of these biopolymers. To overcome these limitations, biomaterials reinforced with natural fibers are acquiring more relevance as the world of bioplastics production is increasing. This review presents an overview of PHA–vegetal fiber composites, the effects of the fiber type, and the production method’s impact on the mechanical, thermal, barrier properties, and biodegradability, all relevant for biopackaging. To acknowledge the behaviors and trends of the biomaterials reinforcement field, we searched for granted patents focusing on bio-packaging applications and gained insight into current industry developments and contributions.

## 1. Introduction

In 2020, the European Parliament approved a strategy for a circular economy in plastics. Some of the main challenges are increasing plastic reuse and recycling rates, achieving a competitive and efficient economy in the use of resources, and undertaking the effort to reduce marine litter [1,2]. The Commission proposal urged to ensure that by 2030 all plastic packaging placed in the European Union (EU) market will be reusable and recyclable in a cost-effective manner, improving the design and collection process and reducing single-use plastic, restricting the use of oxo-degradable products, and defining rules for labeling compostable and biodegradable plastics. It also suggested a list of single-use plastic items to be banned or restricted, such as food and drink containers, drink cups, cutlery, plates, stirrers, bottles, beakers, lids, lightweight plastic carrier bags, and oxo-degradable plastics, among others [3,4]. Oxo-degradable or oxo-biodegradable polymers are mainly petroleum-based products combined with additives to promote fragmentation. Their macromolecular chains split into small chains when these polymers come into contact with heat, oxygen, or light through either biotic or abiotic mechanisms. Examples include low-density polyethylene and polypropylene films combined with metal oxides (Fe_2_O_3_, Cu_x_O, and ZnO) [5,6,7].

Several international organizations such as the Nations Convention on the Law of Seas (UNCLOS) and the International Maritime Organization (IMO) have contributed strategies for preventing, reducing, and controlling pollution from land-based sources, waste from vessels, pollution from the exploitation process, and marine plastic litter from ships [8,9]. At the same time, some countries are leading initiatives to reduce plastic waste. More than 100 governments affirmed their disposition to launch negotiations for a new global plastics agreement in recent years, and almost 127 countries approved legislation to regulate plastic bags and stimulate circular plastic economies. Germany has been named “the champion recycling country” for achieving a 67% recycling rate of household solid waste in 2017, followed by Austria and South Korea with 53%. In addition, China, Malaysia, Vietnam, Thailand, Indonesia, South Korea, Taiwan, India, and Turkey banned plastic waste importation [10,11,12,13]. 

Another factor that increases the urgency of finding solutions to reduce plastic waste is the Chinese prohibition of plastic waste importation. China used to import a large percentage of the global plastic waste for a manually recyclable process. In 2016, China imported approximately 8 million tons of waste from developed countries, and now those governments must recycle their waste or export it to other Asian countries [14,15]. In this context, bioplastics such as polyhydroxyalkanoates (PHAs) have emerged as part of the solution to plastic waste.

Biopackaging, in eco-conscious packaging, is any biodegradable packaging conceived for sustainability. It involves natural and synthetic biodegradable polymers, called biopolymers, that can include by-products of the agro-industry, such as fibers and inorganic or bioactive compounds, to be more respectful to the environment. These materials wrap or contain products temporarily for handling, transport, and storage [16,17,18]. Because biodegradability and biocompatibility are remarkable properties of polyhydroxyalkanoates (PHAs), these polymers are more suited for biopackaging than are synthetic plastics [19].

PHAs are linear and thermoplastic polymers. They can be produced by plants and bacteria such as *Delftia acidovorans*, *Pseudomonas mosselii*, *P. oleovorans*, *P. putida*, *Halomonas* sp., and *Escherichia coli* LS5218 when subjected to stress by lack of nutrients such as nitrogen, phosphorus, and others [20,21,22]. These bacteria store carbon in PHAs granules as an energy reserve [23,24]. PHAs are composed of hydroxyalkanoate monomers susceptible to other bacteria and fungi degradation. For example, microorganisms such as *Paucimonas lemoignei* release depolymerases to degrade the PHA into water-soluble monomers and oligomers [23,25,26]. For example, a PHA bottle can take less than three and a half years to degrade in a marine environment, a short time compared to that required for fossil-derived plastics [25], and a high degradation rate of PHA is achievable in soil (109 days) depending on the soil composition, bacterial population, and crystallinity degree of PHA [27,28].

There are bioplastics made with PHAs already on the market, but despite low-cost applications granted by recently developed extruders and molding machines, the production capacity of PHAs is lower than the current consumption of plastic [29,30]. According to Our World in Data, a project of the Global Change Data Lab, a non-profit organization based in the United Kingdom (Charity Number 1186433), in 2015, approximately 11% of PHA production was used for packaging, although other reports believe it to be 40% [31,32]. While the global capacity for bioplastics production in 2019 was 2.11 million tons, barely 0.57% of the total production of plastics, only 25 thousand tons were PHA (around 1% of total production). The European Bioplastics and Nova-Institute forecasted that by 2024, bioplastic production will be approximately 2.43 million tons, and PHA production will triple the current production [33,34], which is a significant growth but still lower than the current demand for plastics. 

The high production cost of PHAs is another drawback to replacing conventional plastics. This cost comes from the complex production process that includes several steps such as selection of the raw material, bioreaction, separation and drying of the biomass, PHA extraction, and processing [35,36]. The raw material accounts for more than 50% of the production cost [37], whereas the price of PHA can be approximately 300 times the price of a polymer such as polypropylene. Thus, research has focused on selecting cheap raw materials and developing new strategies for PHA production. One of the alternatives to overcome this economic and technical barrier is the elaboration of composites with natural fibers. The vegetal fibers used as fillers are essential in reducing cost, increasing biodegradability, and tailoring mechanical and thermal properties. However, using natural fibers with PHA has limitations, such as low interfacial adhesion to the biopolymer matrix, poor matrix dispersion, and hydrophilic characteristics [38,39,40,41]. 

Some alternatives implemented to surpass the technical disadvantages include making composites with two or more fibers that complement each other, different fiber pretreatments, and compatibilizers [42].

This review aimed to analyze the composites made with PHAs and natural fibers as a realistic alternative for biopackaging.

## 2. PHA–Vegetal Fiber Composites

Biomaterials reinforced with natural fibers are acquiring more relevance as the world of bioplastics production is increasing. The keywords biodegradable, PHAs, fibers, natural, and vegetables were searched in the ISI Web of Knowledge and Scopus, resulting in 87 articles. Below, we discuss the main characteristics required for biocomposites meant for bio-packaging and the impact of the fiber and the preparation method on the composite properties.

### 2.1. Polyhydroxyalkanoate and Fiber Composites

Natural fibers are mainly composed of cellulose, hemicellulose, and lignin, which have different physical and chemical properties [43,44]. Different natural fibers have been used to reinforce PHAs, but their inclusion also presents some issues. For example, cellulose provides the strength of the fiber but has poor compatibility with hydrophobic polymers such as PHA [45,46]. Hemicellulose is amorphous and hydrophilic due to hydroxyl and acetyl groups; therefore, its mechanical properties are poor, and it retains moisture [47,48]. The lining is aromatic and amorphous but less hydrophilic than other components [46,47]. These characteristics cause low interfacial adhesion between fiber and matrix and the generation of polar groups, which generate poor dispersion in the matrix [49,50]. Thus, different pretreatments have been used to reduce the polarity and water absorption of fibers to improve the affinity between the fillers and the matrix and enhance the efficient stress transfer from matrix to fibers. Some examples of the primary pretreatments found in the literature, and the main changes observed in the fibers after the treatment are shown in Table 1. 

With pretreatments of chopping or grinding, fibers are cut by mechanical methods and sieving, or micronized, to obtain smaller particle sizes to improve fiber–matrix adhesion and promote crystallization [51]. Grinding methods include cutting milling, impact milling, or ball milling [52]. Enzymatic pretreatment immerses the fibers in a pectinase, laccase, or cellulase solution to modify the fillers’ surfaces and remove impurities [53]. In grafting, powder cellulose fibers undergo an esterification process and are subsequently dried. These fibers are further treated to increase their hydrophobicity [54]. In mechanical–hydrothermal pretreatment, fibers are immersed in warm water for surface modification and bacterial degradation [55,56]. Mechanical–chemical pretreatment consists of grinding the vegetal fillers followed by an alkali or solvent treatment to remove impurities and to improve the fiber–matrix adhesion [57]. Lastly, an argon plasma jet induces new functional groups on the cellulose surface in the plasma pretreatment, allowing the fibers to ultrasonicate and lyophilize [58]. Additional reports on pretreatment of the fiber with waxes suggest performance improvements without hindering the biodegradability of the composite. For instance, in a blend of PHBV, wheat, ATBC, and calcium carbonate, the pretreatment of the fiber with bio-based waxes improves the mechanical performance of the blend in terms of impact resistance, and in a composite of PHBV–potato–ATBC–calcium carbonate, the wax pretreatment of the fiber enhances the fiber–matrix adhesion and the mechanical properties of the composite [59,60].

**Table 1:** Examples of fiber pretreatments used for biodegradable composite production.

Pretreatment	Natural Fiber	Additive	Treatment Effect	Reference
**Chopped** **grinding**	Wood F		The fibers were too short to hinder the brittle fracture.	[51]
	Basalt F		The fiber–matrix adhesion improved.	[51]
	Rice husk		Irregular fibers in morphology or size	[42]
	*Posidonia oceanica*		Small size; Poor fiber–matrix interaction	[52]
	Cotton		Uniform fiber dispersion due to a grafting process of the polymer matrix.	[53]
**Enzymatic**	Bamboo	Pectinase, cellulase, laccase	Rough surface. Decreased fiber polarity. Better compatibility. Slight increase in tensile and impact strength of composites. Delay in the thermal decomposition of composites. Increased melting peak and crystallization rate. Lower water absorption.	[44]
**Grafted**	Cellulose	Palmitoyl chloride	Increased hydrophobicity. Better fiber distribution in the matrix. Improved elongation of fibers.	[45]
**Mechanical/Hydrothermal**	Ceiba	Immerse in water for bacterial degradation	*	[47]
	Piassava	*	Hemicellulose and lignin partially removed. Increased smooth surface area. Decreased average diameter. Better composites thermal stability.	[54]
**Mechanical/Hydrothermal**	Microcrystalline cellulose	*	Size and shape changed to nanocrystals and nanofibrils. CNC contributed to high and perfect PLLA crystal formation. Composites with CNC have better elongation than composites with MCC.	[55]
**Mechanical–chemical**	Luffa	Alkaline treatment (NaOH)	Hemicelluloses and lignin partially removed. Reduced diameter. Improved adhesion.	[56]
	Olive husk	Acetone–ethanol, NaOH	Hemicellulose and lignin eliminated. Smooth surface. Improved interfacial adhesion.	[57]
	Wheat straw	Alkaline treatment (NaOH)	Non-cellulosic components removed. Straight and smooth surface. Better fiber–matrix interface. Improved tensile strength.	[58]
	Vine shoots	Acetone	Reduction of lignin and resveratrol content. Improved biodegradability of the fiber.	[59]
	Seagrass	Acetic acid to wash, and alkaline treatment (NaOH)	Wax, hemicellulose, lignin, and calcium carbonate impurities removed. More reactive −OH groups in the fiber surface promoted better fiber–matrix interaction.	[60]
	Rice husk	Alkaline treatment (NaOH)	Wax, hemicellulose, and lignin removed. Increased crystallinity.	[60]
	Almond shell	Alkaline treatment (NaOH)	Wax, hemicellulose, and lignin removed. Increased crystallinity.	[60]
	Radiata pine	Sigmacote, hexane, heptane	*	[61]
	Coconut	Oregan essential oil	Fiber length decreased with increasing screw speed. Antibacterial activity (bacteriostatic effect against *S. aureus*).	[48]
	Microcrystalline cellulose		The size was reduced to nano dimensions, and the shape changed to spherical and fibril.	[55]
	Kenaf	Alkaline treatment (NaOH) and silane (triethoxysilyl propylamine)	Improved interfacial fiber–matrix bond but not improved mechanical properties.	[33]
	Palm brunches (Efb)	Alkaline treatment (NaOH) and silane (triethoxysilyl propylamine)	Improved interfacial bond between fibers and matrix but not improved mechanical properties.	[33]
**Plasma**	Microcrystalline cellulose		Surface modification for a better matrix–fiber interface. Decreased thermal stability.	[49]

* Information not provided. Basalt F: basalt fiber; CNC: nanocrystalline cellulose; Efb: empty-fruit palm brunches; MCC: microcrystalline cellulose; NaOH: sodium hydroxide; PLLA: polylactic acid; Wood F: wood fiber.

### 2.2. Mechanical Properties of PHA–Vegetal Fiber Composites

**Figure 1:**
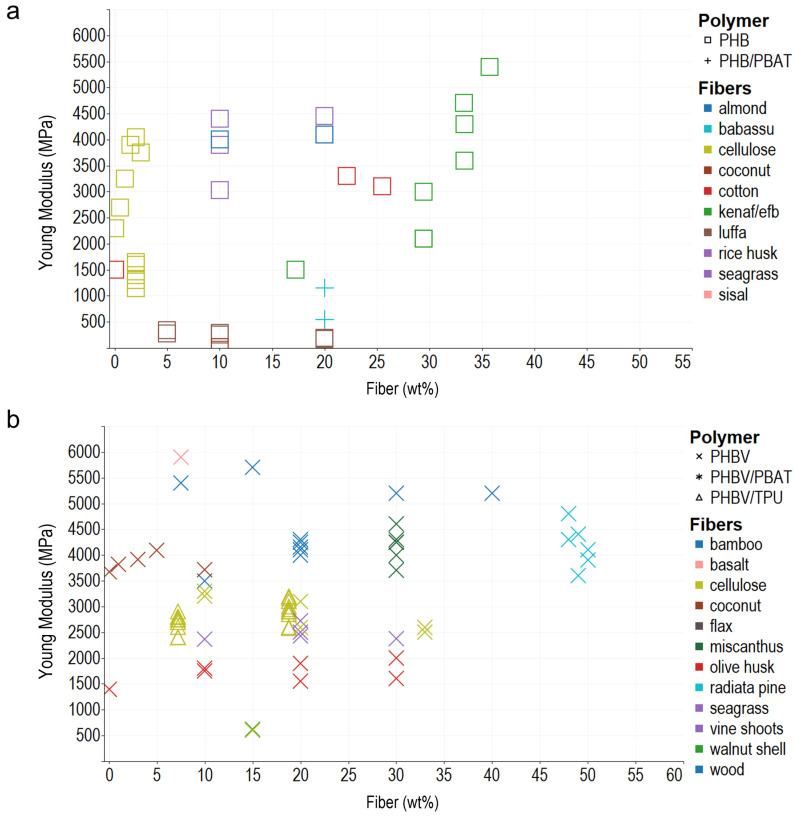
Young modulus of different PHA–fiber composites. (**a**) PHB and PHB/PBAT blends. (**b**) PHBV and its blends with PBAT and TPU. The colors refer to the fiber type (fillers), and the shapes refer to polymer types (matrix).

**Figure 2:**
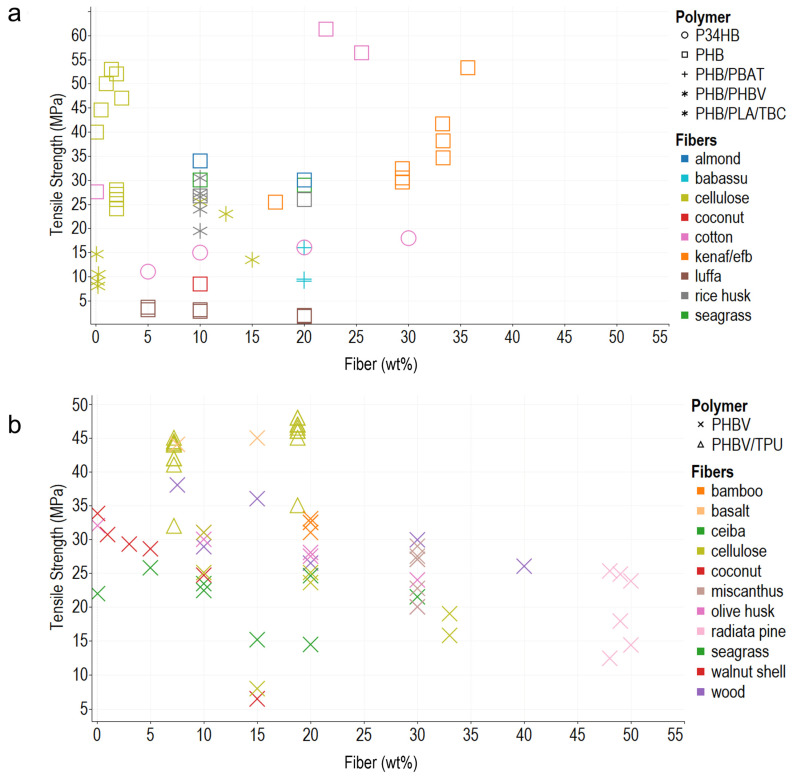
Tensile strength. (**a**) PHB and PHB/PBAT blends. (**b**) PHBV and its blends with PBAT and TPU. The colors refer to the fiber type (fillers), and the shapes refer to polymer types (matrix).

**Figure 3:**
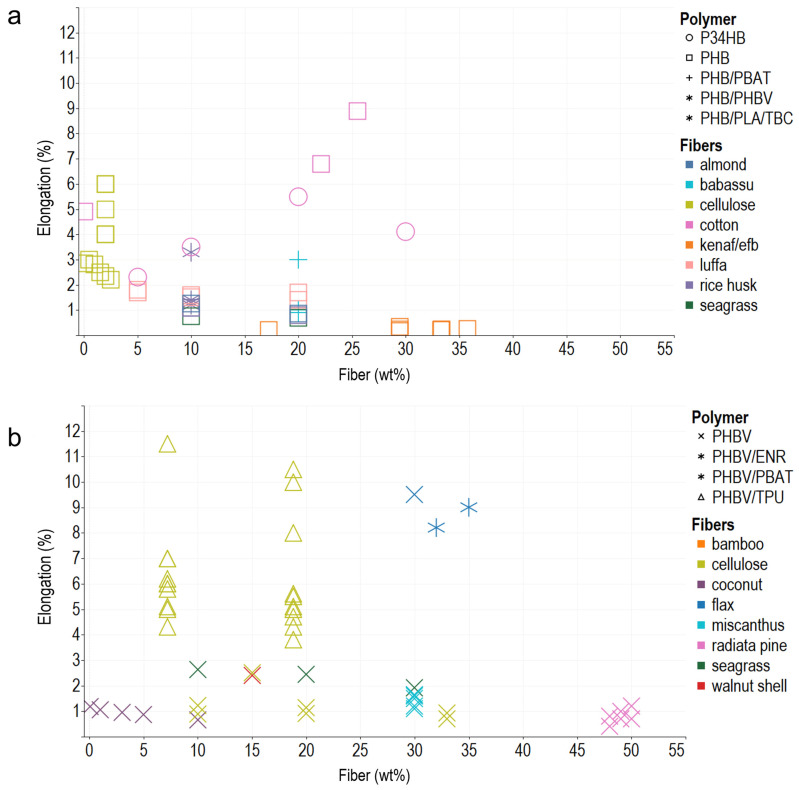
Elongation at break of PHA–fiber composites. (**a**) PHB and its blends with PBAT, PHBV, PLA, and TBC. (**b**) PHBV and its blends with ENR, PBAT, and TPU. The colors refer to the fiber type (fillers), and the shapes refer to polymer types (matrix).

In general, the composite polymers use less than 30 wt% of reinforcements, probably due to the difficulty in achieving a homogeneous dispersion of the vegetable fibers, and melt flow index also decreases, which hinders composites’ processability. Young’s modulus, tensile strength, and elongation at break are the most commonly reported mechanical properties, typically measured using the ASTM D638 Standard Test Method for Tensile Properties of Plastics. In addition, ASTM D790 for Flexural Test of Plastics, ASTM D256 2018 Standard Test Methods for Determining the Impact Resistance of Plastic Izod Pendulum, and ISO 527 Determination of Tensile Properties in Plastic Films are used. After analyzing the literature and organizing the information according to the matrix materials and fibers used as reinforcement in composites, we plotted the reported data, regardless of the preparation method, to acknowledge behaviors and trends of the biomaterial reinforcement using Tableau (Salesforce company) for visual analysis (Figure 1, Figure 2 and Figure 3). 

#### 2.2.1. Young’s Modulus 

The addition of fillers to the polymeric matrix enhances its resistance to stretching or deformation, a property related to the Young’s modulus, independently of the poor filler matrix adhesion (Figure 1). Fibers modify the Young’s modulus, measured in the elastic zone of the material’s stress–strain curve, and the inclusions act as nucleating agents to promote crystallization [55,61]. The Young’s modulus values of some traditional plastics range between 67 and 3100 MPa, and the Young’s modulus of the composites studied in this review exhibited a wide range between 10 and 24,000 MPa [36,62,63]. The fiber type, fiber content, and pretreatment determine the mechanical properties. PHB (polyhydroxybutyrate) and PHBV (polyhydroxybutyrate-co-valerate) fiber composites, PHB/fiber (80/20 wt%) with almond fiber, and rice husk range between 2200 and 2500 MPa. Blends of PHBV bamboo, miscanthus, flax, or wood (70/30 wt%) had Young’s modulus values between 3000 and 5000 MPa. This same range of Young’s modulus values is observed using 50 wt% of radiata fiber or 10 wt% of coconut or cellulose. Nevertheless, incorporating plasticizers into the PHAs matrix reduces Young’s modulus. In a composite of PHBV/TPU(thermoplastic polyurethane)–cellulose, where three different additives were used, the compatibilization between fiber and matrix enhanced and incremented the viscosity of the films, but Young’s modulus decreased compared with the PHBV controls [64,65].

Fiber length impacts the crystal structure. For example, in PLA–PHB–cellulose composites, nanofibers promote crystal size reduction, perfect crystal formation, and homogeneous dispersion, resulting in a stiffer composite [66]. In another example, the addition of short coconut fibers (542–1100 µm) improves crystallinity; however, when fiber content increases, some aggregates are formed in the matrix due to poor dispersion of fibers, which can generate microfractures in the composites [67,68]. In a blend of PHB/PBAT(polybutylene adipate-*co*-terephthalate)–babassu, PHB and PBAT mutually inhibited crystallization, and the resulting composite was amorphous. At room temperature, the increase in PBAT content reduced Young’s modulus compared with PHB film, but when samples were tested at −40 °C, the polymer chain movement was restricted, and the Young’s modulus of the blend increased [69].

The method of obtaining the composites also improves the mechanical properties, providing alternatives to tailoring the elastic modulus of the matrix. Higher Young’s modulus values are obtained when preparing the composite by stacking, casting, extrusion, and injection. For example, the Young’s modulus of a composite of PHBV–wood (85/15 wt%) elaborated by extrusion–injection increased from 4500 MPa (neat PHBV) to 5667 MPa. In a composite with kenaf/Efb, the number of layers and the stiffness of the external layer contribute to increasing the Young’s modulus. Moreover, a PHBV–flax (70/30 wt%) composite made by stacking reached 16.69 GPa, representing a 320% increase compared to the PHBV matrix due to fibers’ properties (flax Young’s modulus is 60–80 GPa). PHA–flax (70/30 wt%), also made by stacking, reached a Young’s modulus of 10.27 GPa, probably due to a reduction of 2% in the porosity. However, when other polymers such as polybutylene adipate *co*-terephthalate (PBAT) or epoxidized natural rubber (ENR) were added to the matrix, the Young’s modulus of the composites was reduced by 15% [48,70]. Although the method of obtaining the composites modifies their mechanical properties, the selection of the method depends on the intended usage of the produced material. For example, casting is ideal for laboratory testing, extrusion is ideal for profile generation, and injection for end products such as containers. The most common methods of composite preparation are listed in Table 2.

#### 2.2.2. Tensile Strength

Tensile strength (UTS) is the maximum point in the stress–strain curve that materials reach without fracture when a load is applied. In composites, the average UTS values range between 20 and 30 MPa, very close or below the UTS of the matrix, mainly due to poor adhesion (Figure 2). The PHBV/ENR–flax (65/35 wt%) composite made by staking achieved the highest UTS value, 188 MPa; PHA–flax reported an 82 MPa, despite the differences in the stiffness of the matrix [70,77]. Fibers properties modulate mechanical behavior. Composite’s UTS depends on many factors such as fiber properties, size, weight, percent of content, processing temperature, and fiber–matrix adhesion [49,88,89]. 

As depicted in Figure 2, the amount of fiber affects the UTS. Some studies report a better performance when adding 1–10 wt% fiber, but the UTS tends to decrease when the content is higher, probably due to the formation of agglomerates [56]. In general, poor compatibility is more evident when filler weight is 30% because fibers act as stress concentrators, and the composites turn into a brittle material, and UTS decreases [80]. Hence, good interfacial adhesion and uniform fiber dispersion are required to improve UTS. Composites can withstand more significant stress before breaking when fibers efficiently transfer stress, which requires pretreatment before the compounding process [79]. Pretreatments aim to enhance fiber–matrix adhesion. For example, the grafting process of PHA and maleic anhydride (MA) used to reduce the hydrophilicity of composites had a positive effect on mechanical properties [73]. In addition, in a PHBV–bamboo composite, the enzymatic treatment leads to better fiber bonding and a UTS increase of 4% [53]. Likewise, plasma treatment in PHB–cellulose (98–2 wt%) increases the UTS by 15% [58].

The fiber size also affects the UTS. For example, in a PHBV–coconut fiber composite made by extrusion–compression molding, high long fiber content hinders chain-folding and the formation of the crystals, but high short fiber content leads to a nucleating effect, which results in a lower UTS for the blend [57]. Thus, fiber length is critical for the efficiency of the reinforcement. In a composite of PHB–sisal fibers, long fibers increase the blend’s moisture content, affecting the interfacial interaction, which causes a reduction in the UTS from 22.3 to 11.9 and 18.2 MPa [76]. When fiber size appears not to affect the UTS, it is probably due to variability, which masks the fiber type and size effect [90].

#### 2.2.3. Elongation at Break

Elongation at break is a differential between the initial longitude and the final longitude of a specimen after the breakage test, generally expressed as a percentage. The elongation at break is poor in fragile materials due to large spherulites generating processing gaps, and the addition of vegetal fillers to a matrix impacts the net’s elongation. Figure 3 shows the relationship between fiber percentage and elongation at break. The elongation at break is lower than 7% in most composites. Contrary to Young’s modulus, fiber addition leads to a reduction in elongation at break. The PHA–chestnut (90/10 wt%) composite made by compression molding has an elongation at break of 580%, the highest value reported (not depicted in Figure 3). In this composite, grafting with glycidyl methacrylate (GMA) contributed to more uniformly dispersed fibers, better wetting, and improved interfacial adhesion due to the similar hydrophilicity of the phases [91].

Typically, fibers compromise the elongation at break. Long fibers promote the formation of large crystals and, therefore, a more fragile material, whereas the crystals formed are smaller when short fibers are used [72]. In addition, fiber orientation may cause stiffness and reduce the flexibility of biopolymer nets [77]. However, incorporating plasticizers into the blends promotes fiber–matrix bonding. For instance, in the PHBV/TPU–cellulose blend produced by extrusion–injection, incorporating 1 phr of hexamethylene diisocyanate (HMDI) enhances the interfacial adhesion and improves the composite elongation by 150% [64]. The addition of ATBC increased the elongation at break to 6.4%, triple that of PHBV film [52]. Nonetheless, in some cases, the fiber content’s effect exceeds the plasticizer’s effect, as in the composite PHBV–posidonia (70/30 wt%), where the ATBC could not compensate the fiber’s stiff effect [83]. In a composite made by lamination, the joncryl additive partially fills the gap between fiber and matrix but does not affect crystallization or elongation at break [87].

### 2.3. Thermal Properties of PHA–Fiber Composites

Two main techniques determine transition temperatures to characterize polymers. In differential scanning calorimetry (DSC), the specimens are subjected to two heating cycles. The first heating process has the objective to erase the thermal history of the polymer matrix and remove moisture because water acts as a plasticizer and modifies the properties of the polymers, and the second cycle identifies melting and crystallinity temperature, and, in some cases, the generation of crystals of different sizes [92,93]. Thermogravimetric analysis (TGA) measures the mass variation when the temperature changes [94,95].

**Figure 4:**
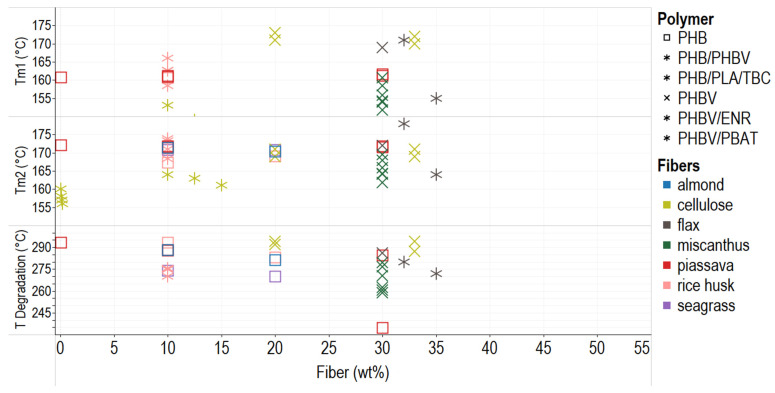
Thermal behavior of PHA–fiber composites. The colors refer to the fiber type (fillers), and the shapes refer to polymer types (matrix). Tm = temperature of melting.

Figure 4 shows the relationship between fiber percentage and melting and degradation temperatures. Fiber addition does not significantly modify the material’s melting temperature, as seen in the compounds PHB–pissaba, PHB–rice husk, and PHBV–cellulose. This behavior is desirable since inexpensive vegetable fillers can be used in composites to reduce the cost without significantly affecting processability. Thermal stability is critical for packaging applications because some containers are exposed to high or low temperatures during shipping and storage. A biocomposite must endure heating or cooling processes [96,97,98].

The TGA shows that the degradation of composites PHA-vegetal fibers occurs in two main steps: first, the initial fiber and PHA degradation by hydrolysis, and second, the lining and residue degradation at 350 °C or more [99,100]. The addition of fibers also implies the addition of impurities, and the initial temperature of degradation (Tdeg) decreases; also, some interaction between fibers and PHBV matrix results in lower Tdeg of composites [101]. Fiber addition also causes differential melting temperatures in the first heating (Tm1) and the second cycle (Tm2) due to perfect crystal formation because more giant and more ordered crystals need more energy to melt again [51,84]. This behavior has been reported for PHB–flax, PHBV/ENR–flax, or PHBV–miscanthus composites (Figure 4). The degradation temperature of the composites decreases when using more significant amounts of the filler and additional treatments to reinforce, which implies less thermal stability.

Some treatments improve the thermal stability of composites due to the removal of pectin, cellulose, and other substances of the filler [78]; when fiber improves the interaction with the matrix, the thermal degradation is retarded [54]. Likewise, reactive agents impact the thermal behavior of composites. For example, DCP (>0.1 phr), the additive in a blend of PHBV–miscanthus (70–30 wt%). reduces the temperature of melting (T_m_) of the blend by reducing crystallinity [65]. In some cases, additives mask the nucleating effect of the vegetable fiber in the polymeric matrix. In addition, plasticizers, typically used for internal lubrication, increase mobility and decrease the temperature of glass transition (T_g_) [72]. The loss of the plasticizer usually appears in the first part of a TGA curve. This behavior is typical in PHB composites with glycerol and triethyl citrate (TEC), among others [76].

### 2.4. Barrier Properties of PHA–Fiber Composites

The barrier properties are essential for packaging materials, especially in food and shelf applications [102,103]. If a composite absorbs water or oils or has a high gas and vapor permeability, it is unsuitable for preserving the organoleptic properties of the package content [104]. The addition of vegetal fibers to a polymer matrix increases the porosity and the number of polar groups that result in absorbing water. Moreover, poor interfacial adhesion creates zones that efficiently uptake water [85,105]. The moisture reduces the mechanical properties of the blend and increases biodegradation because the water migrates to the amorphous zones and leads to polymer chain scission. Pretreatment of the vegetable fibers would reduce moisture absorption. Pretreatments such as esterification, use of NaOH, or enzymatic reduction achieve better dispersion and adhesion of the filler into the matrix, reducing the hygroscopicity [78,106].

Noteworthily, the water vapor transmission (WVP) of PHA films is similar to polyethyleneterephtalate (PET) films [104], but it increases with the addition of vegetal fibers because of the crystallinity changes generated by the fillers [107]. The WVP of a composite, as a measure of water vapor uptake, depends on fiber amount, crystallinity decrement, and changes in the molecular weight of the matrix [54]. The type of fiber and its hygroscopicity also affects the water vapor permeability [74]. For example, adding low amounts of fiber (2%) in a PHB–cellulose composite improves the crystallinity, reducing the diffusion process.

### 2.5. Biodegradability

A biodegradable polymer undergoes biodegradation, a chemical process during which microorganisms that are available in the environment decompose materials into natural substances such as water, carbon dioxide, and methane [108]. As per the ASTM D6400 definition, compostable plastics must demonstrate proper disintegration during the composting, an adequate level of inherent biodegradation, and no adverse impacts to support plant growth [109]. Most materials are biodegradable, but their biodegradation process might take hundreds of years [110]. Therefore, one of the objectives of biodegradable plastic developers is to achieve this process within an appropriate life span according to the use of the material and its subsequent disposal.

There are different methods to measure a polymer’s biodegradability. The test selection depends on the organization (ASTM, EPA, ODEC, ISO), external conditions (aerobic, anaerobic, UV exposure), the environment (soil, marine water, compost), or their purpose (biodeterioration, assimilation, biofragmentation) [111,112]. Among the most common tests used to monitor biodegradation are weight loss, abiotic degradation, CO_2_ measurement, macromolecular weight loss, oxygen consumption rate, and anaerobic digestion (biogas production–weight loss) in compliance with ISO 15814:1999, ISO 17556 (2019), ASTM G160–12, and ASTM D6691 standards [113,114,115,116,117]. CO_2_ measurements with a respirometric test also help identify the material’s shelf life according to ASTM D5988-96. These standards require that the material biodegrades in a certain period and leaves no toxic residue in the soil. Exposure to the environment (i.e., temperature, moisture, microbial population, pH, oxygen content) affects the biodegradation of a polymer; thus, a material that degrades by microbial activity under industrial composting conditions may not degrade in other conditions [118].

Reinforcement with vegetal fibers is expected to improve the biodegradability of the already biodegradable PHAs. Instead, biodegradation depends on soil composition, fiber amount and pretreatment, material stiffness, and processing [85]. For instance, the PHBV–shoot vine composite biodegradation rate is 83%. The content of lignin and polyphenols makes biodegradation difficult, but pretreatment of the fibers raises the composite’s biodegradation to 97%. In a different example, PHB degradation ranged from 60 to 98%, depending on the method. PHB showed 64.3% degradation in 6 months using microbial fermentation in soil tests [119]. In a quasi-steady state, co-digestion of synthetic municipal primary sludge (SMWS) and PHB, after 45 days, exhibited approximately 80–98% conversion of PHB to biomethane [120]. In soil, P(3HB) specimens had been 60% degraded in 21 days, but they continued to degrade to 93% by day 35 [121]. For comparison, blending PHB with wood fiber yields conflicting results. The biodegradation of [P(3HB-*co*-3HHx)]/Kenaf during 48 days in mineral medium-soil reached 13%, whereas, in an aqueous-nutrient medium, it barely reached 2.4% [113]. Biodegradation of a composite of P(3HB-*co*-4HB)/wood under laboratory composting for 21 days could not be detected, but in an aqueous medium, it was 0.35% in two months. However, biodegradation was 35% per year in soil [114,116].

Additives also impact biodegradability. TPU (18–21 wt%) added to a PHBV–cellulose composite reduced the disintegration of the blend because the TPU covered the filler and interfered with the microorganism’s access. When using HMDI, the degradation rate increased because the plasticizer blocked the effect of the TPU on the fibers [74]. Likewise, bio-based plastics with additives tested in soil media for 660 days did not show significant biodegradation. Instead, the PHA film reported 70% mineralization, very similar to the cellulose control film measured under the same conditions [122]. In a PHBV–posidonia composite, using ATBC as a plasticizer increases the polymer chain mobility and accelerates the disintegration of the blend [83]. This research measured the specimen’s degradation in marine mesocosms and found degradation in warm seawater conditions. The specimens used in the test maintained their tensile properties by ten months, suggesting possible applications in marine ítems.

Disposal in landfills raises additional concerns for biodegradable plastics. A food packaging study observed that packaging made with biodegradable materials releases methane, more harmful than CO_2_ [123]. Thus, alternate bioremediation strategies are needed, such as using methanotrophic microorganisms to reduce methane emissions in landfills or using the methane for energy generation [124,125,126] to take full advantage of biopackaging waste biodegradation.

### 2.6. Theoretical Modeling to Evaluate Performance and Applications of Polymer–Vegetal Fiber Composites

Although blending with fibers improves the polymers’ mechanical properties, the extent of the fiber contribution is unknown. Theoretical modeling has helped infer the performance and modifications expected for these composites. PHA/hemp and PHA/jute (30 wt%) modeling showed damage on the matrix due to the different physical properties of the fillers. In this study, hemp was the best filler and achieved a better interface that supports higher mechanical loads [127]. A PHBV/oak wood flour composite was modeled using a modified Fickian diffusion law and the Halping-Tsai and Nicolas and Nicodemo model to predict composite properties, such as moisture absorption, stiffness, and strength. Despite the improved mechanical properties of the composite, the blend is susceptible to deterioration by the hygrothermal behavior of the fiber [128].

Further modeling and experimental testing are essential to make better predictions. A 3D model of PHBV–wheat straw designed to predict water vapor permeability using the finite element method (FEM) to the 3D structures permitted a better prediction of water vapor permeability dynamics of the composite [129]. Numerical homogenization and representative volume elements (RVEs) are used to model composites’ effective elastic, thermal, and thermoelastic properties. This methodology allows for the preservation of fiber–matrix interactions and the predicted effective properties of blends, which can be further validated with experimental data [130].

## 3. PHAs Composite Applications in Packaging: Contributions of the Industry

Researchers have arduously worked on designing and preparing biocomposites, and one of the leading applications proposed for PHA–vegetal fiber composites is bio-packaging. Nevertheless, not all of the composites have possible industrial applicability. To determine the extent of industrial applicability of PHA–vegetal fiber composites, we searched for patents in the Lens database and Web of Science. After eliminating medical, veterinary, processing methods, machines, or equipment, we found 141 patents. The patents we found belong to the subgroup C08L6731, according to the Cooperative Patent Classification (CPC) system, comprising compositions of polyesters obtained by reactions that form a carboxylic ester bond in the main chain of polyester-amides, the subgroup CO8L2666, comprising polymers characterized by an additional compound in the mixture, and the subgroup Y10T428, comprising stock materials or miscellaneous.

The patents’ owners are developing materials to adjust to the new normativity demanding reduction of the environmental impact. The inventions are summarized in Table 3. The patents offer materials with a wide range of applications, and biodegradability is paramount in these composites. Most patents related to food packaging acknowledge that PHAs are safe for food and contribute to preserving their organoleptic properties. Some materials can preserve and protect against contamination with bacteria and fungi [131], and others keep the product’s aroma, flavor, and texture [132]. Most of the products are designed to serve as films for molding or coating. Others are ideal for stacking, where different layers complement each other. PHAs are brittle and stiff, but a layer of PLA, PLC, or PBS and a plasticizer reinforce the processability of the blend.

The relevance of biocomposites, particularly PHA–vegetal fiber composites, in many applications has attracted the industry’s attention, mainly because fillers also reduce composite costs. Independently of their business sector, important companies invest in developing blends with PHAs. Some chemical companies such as DuPont, BASF, Cargill-Dow Polymers, Union Carbide, Bayer, Monsanto, Mitsui, and Eastman Chemical developed and currently sell biodegradable products. Among them are blends of PLA, PET, or PBAT, additives to enhance PLA properties, or even products ideal for blending with fibers such as ECOFLEX/ECOVIO and EASTAR BIO aliphatic–aromatic polymers, BAK (a polyester amide_, and BIOMAX for modified PET and PLA [133,134].

**Table 3:** Patents of materials with PHAs and natural fibers for packaging or similar applications.

Year	Publication Title	Owner	Matrix/Base	Filler/Additives	Applications	Publication Number	Group	Ref
2021	Composite materials, methods of making, methods of use, and articles incorporating the composite materials	NIKE INC			Articles that undergo water contact	US 10919257 B2	B32B *	[135]
2020	Film packaging for oral biologics	CEVA SANTE ANIMALE S A	Biodegradable polymers and petroleum-based polymers		Films for packaging oral biologics such as vaccines.	EP 2775986 B1	A61J, A61K, B32B, B65D *	[136]
	Compositions containing new polyesters	NOVAMONT SPA	Polyester, PHAs, aliphatic, and/or aromatic polyesters.		Mass-produced articles	US 10738149 B2	C08K, C08G, C08J, C08K, C08K, C08L *	[137]
	Biodegradable sheets	TIPA CORP LTD	PBS, PBAT, PHA, PLA		Biodegradable sheets	US 10675845 B2	B32B, C08K, C08L, C09D *	[138]
2019	Biodegradable fabric and methods of making and using the same	SANCTUARY SYSTEM LLC	PLA		Packaging material, health care articles, and household products	WO2019070633	D06M	
	Biodegradable sheet	TIPA CORP LTD	PBS, PBAT, PHA, PLA		Biodegradable sheets	US 10239292 B2	C08L, B32B, C08J, C08K,	[139]
2016	Bio-based modifiers for polyvinylchloride blends	METABOLIX INC	Polyvinylchloride (PVC) and PHA		Packaging	US 9505927 B2	B32B, B29C, Y10T	[140]
	Biodegradable polymer films and sheets suitable for use as laminate coatings as well as wraps and other packaging materials	BIOTEC BIOLOG NATURVERPACK	Polyester amides and other polyesters, and natural polymers	Inorganic fillers and plasticizers	Packaging, coating, and wrapping for fast food	EP 2357209 B1	D21H, B29C, B29K, B42D, C08J, C08L, D21D, D21H, Y10T	[141]
	Multilayer article comprising a biodegradable polymer-based layer and a cellulose–fiber-based support; method of manufacturing multilayer article and food accessory comprising a multilayer article	AHLSTROM OY	PHAs, PLA, polybutylene succinate (PBS), biopolyesters	Non-woven fiber layer, kraft, and parchment, food-safe adhesive	Food molds resistant to moisture, food accessory agro-food industry.	EP 2841263 B1	B32B, Y10T	[142]
	Process for manufacturing a composite article comprising cellulose pulp fibers and a thermoplastic matrix	SÖDRA SKOGSÄGARNA EKONOMISK FÖRENING	Polyolefins, PHAs, PLA, polycarbonates, polyvinyl, and mixtures thereof	Cellulose pulp fibers and lubricant	Food containers and packaging	EP 2847382 B1	D21H, B32B, B226, Y10T	
2015	Film with compostable heat seal layer	FRITO-LAY NORTH AMERICA INC/MOUNT III ELDRIDGE M; PALTA DEEPALI	PHBV, PHA		Flexible packaging film with heat seal layer	US 9162421 B2	C09J, C08K, C08L, C09J	[132]

* Information not provided. CPC classes: A61L, methods or utensils to sterilize material or objects, B, performing operations; C08J, composites processed after treatment; C08K, use of inorganic substances as composite ingredients; C08L, organic macromolecular compounds and their preparation; C09J, use of materials as adhesives; D21D, treatment of materials for papermaking; Y10T, technical subjects covered by former US classification.

## 4. Conclusions

Research on biodegradable polymers and vegetal inclusions has grown significantly in the last ten tears. Furthermore, the increasing patents of materials using PHAs highlight these biocomposites’ roles in replacing fossil-derived plastics. This review identified different alternatives to tailoring composite properties. It is possible to use vegetal fibers to enhance Young’s modulus, but that generates poor elongation at break and less thermal stability. Plasticizers enhance the composite elongation, but the degradation rate tends to decrease. Therefore, it is imperative to define objectives and applications before selecting the methodology to produce the blends. Designing a material that meets usage requirements without sacrificing quality standards is essential to compete with conventional plastics.

Most of the publications reviewed above claim that their new composite is ideal for bio-packaging, but the usability of the material is rarely evaluated. Usability tests should be implemented in the industrial environment to evaluate the interactions of the polymers with different food types and conditions. In addition, it would be interesting to incorporate antimicrobial compounds that prolong the product’s life. Researchers have a worthy challenge in designing biopolymers and compounds suitable for packaging applications and exploring other possible uses such as tissue regeneration, plant growth, and automotive applications. Prospects also include designing and manufacturing materials composed of PHA and local agricultural by-products, characterizing them, and evaluating the possibility of using them in food packaging, potentializing at the same time the local circular economy.

## Figures and Tables

**Table 2:** Common methods to produce biodegradable composites.

Process	Matrix	Fiber	Additive	Applications	Reference
**Casting**	PHB	Luffa	*	Packaging	[71]
	PHBV	Ceiba	*	Fresh fruit packaging	[56]
	PLLA, PHB	MCC	Tributyl citrate	Food packaging	[72]
	P34HB	Cotton	Benzoyl peroxide and maleic anhydride.	Paper-based packing	[73]
**Compression**	PHB	Seagrass, almond shell, rice husk		Food packaging	[74]
	PHB	Microcrystalline cellulose	*	Biomedical and engineering uses	[58]
	PHBV	Radiata pine	Polymethylene diphenyl diisocyanate	*	[75]
	PHB	Coconut, sisal	Glycerol	Small tubes and plastic bags for planting	[76]
**Extrusion–compression**	PHB	Wheat straw	*	Biomedical and food packaging, biodegradable polymer	[77]
	PHBV	Bamboo, luffa	*	*	[78]
					
	PHBV	Rice husk	TGIC, DCP	Food packaging	[51]
	PHBV	Coconut		Food packaging	[57]
**Extrusion–injection**	PHBV	Bamboo	*	*	[53]
	PHBV	Olive husk	*	Environmentally-friendly material	[79]
	PHBV	Radiata pine	*	Improved mechanical properties of PHBV composites	[80]
	PHBV	Wood/basalt	*	Long-life material products	[81]
	PHBV	Cellulose	*	Biocomposites with tailored properties	[54]
	PHB	Piassaba	*	*	[66]
	PHBV	Posidonia oceanica	*	Bio-container for plants	[82]
	PHBV, TPU	Cellulose	Hexamethylene diisocyanate, joncryl, TGIC.	*	[64]
	PHB, PBAT	Babassu	*	Several applications	[69]
	PHBV	Posidonia oceanica	ATBC	Seawater applications.	[83]
**Extrusion–injection**	PHBV	Miscanthus	DCP	*	[65]
**Injection**	PHBV	Nanocellulose, walnut, eggshell, tuff	*	Packaging for airline cosmetics food	[84]
**Micro-compounding**	PHBV	Vine shoots		Biodegradable materials	[85]
**Stacking–compression**	PHA, PLA, PBS, PP	Flax	*	Adjustable mechanical properties for industrial products	[86]
	PHBV, PBAT, ENR	Flax	Epoxy sizing	*	[70]
	PHB, PLA	Cotton	*	Building, furniture, or automotive products	[87]
	PHB	Efb, kenaf bast fiber	Triethyl citrate	Replacement of wood products	[42]

Processing conditions such as temperature, time, speed, or pressure were different for each report. * Information not provided. ATBC: acetyl tributyl citrate; DCP: dicumyl peroxide; Efb: empty-fruit palm brunches; ENR: epoxidized natural rubber; GMA: glycidyl methacrylate; MCC: microcrystalline cellulose; P34HB: poly-3-hydroxybutyrate-*co*-4-hydroxybutyrate; PBAT: polybutylene adipate-*co*-terephthalate; PBS: polybutylene succinate; PHA: polyhydroxyalkanoate; PHB or P3HB: poly(3-hydroxybutyrate); PHBV: polyhydroxyburytrate co-valerate); PLA: polylactic acid; PLLA: poly-l-lactide; PP: polypropylene; TGIC: triglycidyl isocyanurate; TPU: thermoplastic polyurethane.

## Data Availability

The data used to support the findings of this study are included within the article.
